# A Systematic Review of Cancel Culture Induced Anxiety (CCIA) and Its Psychological and Behavioural Effects on Gen Z

**DOI:** 10.1192/bjo.2026.11186

**Published:** 2026-06-30

**Authors:** Tolu Afelumo, Simpson Wong

**Affiliations:** 1 King’s College London, London, United Kingdom; 2 Queen Mary University of London, London, United Kingdom

## Abstract

**Aims:**

This systematic review investigates the psychological phenomenon of Cancel Culture Induced Anxiety (CCIA) among Generation Z individuals aged 16–25. The primary aims are to synthesise empirical evidence from existing literature and develop an integrated conceptual model mapping the causes, pathways, and psychological effects of this condition.

**Methods:**

A systematic review was conducted in accordance with PRISMA guidelines and the Cochrane Method. Comprehensive literature searches were performed across PsychINFO,PubMed, and Scopus to identify peer-reviewed, primary empirical studies. Ten studies were selected for inclusion based on a predefined Population, Exposure, Outcome (PEO) framework. Quality Assessment was then performed using Critical Appraisal Skills Programme (CASP) checklists. Finally, evidence was synthesised using the Temporal Need Threat Model, context collapse, and networked publics as theoretical foundations.

**Results:**

Findings indicate that CCIA manifests primarily as acute social anxiety, which frequently escalates to clinical depression, diminished self-esteem, and in severe cases, suicidal ideation. Key behavioural outcomes include avoidance, self-censorship, and both internal and external conformity. These effects are sustained through psychological mechanisms such as rejection sensitivity and rumination. The review establishes that CCIA is driven by simultaneous threats to four fundamental human needs: belonging, self-esteem, control and meaningful existence. Social media, shaped by the dynamics of context collapse, emerges as the dominant environment for CCIA, though its severity is mediated by cultural, socio-economic, and individual predisposing and protective factors. The identified coping strategies are predominantly reactive and individualised, highlighting a significant gap for systemic and preventative interventions.

**Conclusion:**

The proposed conceptual model establishes a vital foundation for understanding CCIA across diverse cultural and social contexts. It offers practical guidance for psychiatrists and mental health professionals in designing targeted clinical interventions and institutional policies. Future research should employ longitudinal and mixed-method approaches to validate the model and inform preventative strategies, addressing CCIA as a systemic feature of the digital age.

